# Plant cognition after Darwin: historical and epistemological remarks

**DOI:** 10.1007/s40656-026-00739-0

**Published:** 2026-06-24

**Authors:** Paolo Pecere

**Affiliations:** Department of Philosophy, Communication and Performing Arts, University of Roma Tre, Via Ostiense, 234-236, 00154 Rome, Italy

**Keywords:** Cognition, Intelligence, Sentience, Evolution

## Abstract

Plant “intelligence” and “sensation” are controversial notions in contemporary botany. While some scientists argue that sensory awareness, learning, memory, communication, and possibly a kind of consciousness should be ascribed to plants, a substantial part of the scientific community dismisses such claims because of insufficient evidence and the lack of neural systems in plants. In this paper, I will examine this issue from a historical and epistemological perspective. First, I will go back to a crucial precedent that is often evoked in these debates, i.e., Darwin’s remarks on plant “sensation” and “behavior” and his analogy between roots and brains in the 1880 book The Power of Movement in Plants. I will point out that the issue was already well known and controversial in the nineteenth century. I will then examine contemporary debates starting from new experiments and evidence on plant cognitive powers. I will argue that current debates are partly based on elements that have been available since Darwin, such as postulates of biological continuity among living beings and the use of analogy, leading to an epistemological contrast between the need to avoid ungrounded anthropomorphic projections and the argument that kinds of cognitive states could be realized in plants. I also finally point out that investigations on animal ethics and the revaluation of non-Western views on animism and ecology have recently been introduced into this debate to break the epistemological balance between opposing views.

## Introduction

Plant cognition is a controversial issue in contemporary botany and in the philosophy of life sciences. A number of scientists have argued that sensory awareness, learning, memory, communication, and possibly a kind of consciousness should be ascribed to plants. A substantial part of the scientific community has rejected their claims because of paucity of evidence and the lack of neural systems in plants, although further empirical investigations are ongoing. In light of new evidence, the debate concerns the attribution of different notions and capacities to plants, ranging from “intelligence” to “sensation”. This conceptual side of the investigation raises an epistemological question: how far can we legitimately stretch our analogies concerning the “mental” life of plants?

In this paper, I will examine, this issue in historical perspective, thereby aiming to contribute to a broader epistemological framework for investigating this controversy. I will start from Charles and Francis Darwin’s remarks on plant “sensation” and “behavior”, and their analogy between roots and brains in the 1880 book *The Power of Movement in Plants*. I will show that the issue was already well-known and controversial in the nineteenth century, as evolutionary theory incorporated previous physiological notions such as irritability and scientists were already divided on the attribution of mental capacities to plants, with positions ranging from reductive materialism to agnosticism and panpsychism.

Against this background, I will examine contemporary observations and experiments on plant cognitive powers together with the corresponding epistemological debates. I will identify a recurring epistemological contrast between the need to avoid ungrounded anthropomorphic projections and the possibility that kinds of mental states exist in plants. In addition, I will point out that new investigations on animal minds and ethics, and a revaluation of non-Western views on ecology have been recently introduced into this debate to upset the established epistemological balance.

## Darwin’s “breakthrough” and its reappraisal

In the final chapter of their 1880 book *The Power of Movement in Plants*, Charles and Francis Darwin formulated a famous analogy between the tip of radicles and brains:

“We believe that there is no structure in plants more wonderful, as far as its *functions* are concerned, than the tip of the radicle. If the tip be lightly pressed or burnt or cut, it *transmits an influence to the upper adjoining part*, causing it to bend away from the affected side; and, what is more surprising, the tip can distinguish between a slightly harder and softer object, by which it is simultaneously pressed on opposite sides […]. In almost every case we can clearly perceive the *final purpose* or advantage of the several movements. Two, or perhaps more, of the exciting causes often act simultaneously on the tip, and one conquers the other, no doubt in accordance with its importance for the life of the plant. The course pursued by the radicle in penetrating the ground must be determined by the tip; hence it has acquired such diverse *kinds of sensitivities*. It is hardly an exaggeration to say that the tip of the radicle thus endowed, and having the power of *directing the movements* of the adjoining parts, acts *like the brain of one of the lower animals*; the brain being seated within the anterior end of the body; receiving impressions from the sense-organs, and directing the several movements” (Darwin & Darwin, 1880, pp. 572–573, my italics).

In this seminal text, the Darwins develop a progressive succession of concepts of capacities: functions, physiological transmission and purposefulness belong to a standard set of properties of living beings in modern life sciences. The attribution of “kinds of sensitivities” and the analogy of roots with the animal brain form a more excentric pair of notions: while similar ideas were widespread in the philosophical-scientific tradition since antiquity,^1^ the attribution of sensitivity to organisms without nervous system was (and is still) at odds with contemporary standards. Indeed, scientists who are recently reconsidering such ideas present Darwin’s views as a valuable theoretical precedent. As František Baluška, Stefano Mancuso and their co-authors write in a programmatic paper, *The Power of Movement in Plants.*

“represents a breakthrough in plant biology. In this revolutionary book, the Darwins departed from the classical and still dominant view of plants as organisms which had no need of movements that were based on *sensory perceptions* or a *brain-like organ*” (Baluška et al., 2009, p. 1121, my italics).

According to the authors, new evidence suggests that this pioneering view of plants as capable of a kind of sensory perception needs to be reevaluated:

“Present day results […] are increasingly coming to show that, in contrast with the classical view, plants are definitely not passive automatic organisms. On the contrary, they possess a *sensory-based cognition* which leads to *behavior*, *decisions* and even displays of *prototypic intelligence*” (Ibid., my italics).

This claim is based on evidence that root caps orient growth in directions that respond to environmental conditions (such as the distribution of resources and the presence of physical obstacles and biological dangers), whereas the severing of root caps is followed by growth in the previous direction regardless of conditions and obstacles. On the basis of this evidence, these authors associate a range of notions to Darwin’s influential account of plant cognition – from sensitivity to decision, behavior, and communication – arguing for the legitimacy of an analogy with animals that culminates in the attribution of brain-like functions to vegetal tissues.

“In keeping with Charles Darwin’s theory of common descent of all organisms, a unification of animals/humans and plants according to their body polarity is possible and thereby *removes from view the Aristotelian dichotomy between plant and animal organisms*, an axiom of classical biology which has posed a serious problem in the understanding of the logic of evolution and the nature of biological systems. The common descent of all organisms is the central pillar of Charles Darwin’s theory of evolution and, as pledged by Sir Jagadish Chandra Bose, the *unity of life* implied thereby is a revelation of both beauty and simplicity. By the same token, the existence of a plant neurobiology harmonises with the neurobiology of animals” (Ibid., p. 1125).

In order to examine this reference to Darwinian biology, we need to consider which theses were actually defended by Darwin in the context of nineteenth-century life sciences and philosophy.

## Irritability vs sentience of plants: a modern debate

Darwin’s views on plants’ cognitive powers, however based on extensive observations and experiments and unorthodox in the context of his time, were not entirely new. In fact, Darwin reprised the modern dichotomy between two competing views: the merely physiological notion of “irritation” and the attribution of full-fledged “sensation” (or sensitiveness) to plants. Both notions appear in different passages of *The Power of Movement in Plants*:

“Various trials were made for ascertaining, as far as we could, the nature and degree of *irritation* to which the apex must be subjected, in order that the terminal growing part should bend away, as if to avoid the cause of irritation” (Darwin & Darwin, 1880, p. 146, my italics).

“We were therefore led to suspect that the apex was *sensitive to contact*, and that an effect was transmitted from it to the upper part of the radicle, which was thus excited to bend away from the touching object” (Ibid., p. 131, my italics).

The alternative between irritability and sensibility of living tissues has a long tradition in modern philosophy and physiology.^2^ Irritability was originally defined by Francis Glisson to account for movements of living beings that cannot be explained mechanically and apparently require perception.^3^ Albrecht von Haller credited Glisson for the discovery of the “active force of our bodies” and the invention of the word *irritability*, but he rejected Glisson’s view that irritability is grounded on *unconscious perception*, claiming that the latter notion was contradictory. Haller maintained that irritability was a basic property of living matter displayed by phenomena, analogous to gravity, whose cause “lies concealed beyond the reach of the knife and microscope” (Haller, 1936, p. 8). While Glisson had a metaphysical view of irritability as a property of matter, Haller included irritability among three “reactive” forces defined by induction: elasticity, irritability (reaction without feeling), and sensation or feeling – the latter being a power of consciousness located in the nerves (Haller, 1757–1766, pp. IV, 467–470). Both authors agreed that a specific power of irritability was needed to explain reactions of living tissues to given stimuli (Giglioni, 2008).

An important turn in this debate was taken by Alexander von Humboldt (who was a prominent scientific model for Darwin). Humboldt’s position illustrates an epistemological division concerning the powers of plants that was much debated in Darwin’s times. Humboldt had defended the need for a specific “vital force” in his early work on plants, the 1793 *Florae fribergensis specimen*. In this study, he tried to apply Lavoisier’s chemistry to plant physiology. Humboldt also raised the question whether plants, although they lack nerves, might be endowed with sensibility, concluding with a cautious remark.

“So far, we see no nerves in plant bodies, and it is claimed that the idea of sentience is related exclusively to nerves. Despite this, however, we are unable to settle the controversy, which has begun among philosophers since ancient times, as to whether plants are truly sentient. This is, in fact, a purely subjective question, of which, beyond the proper sense, no sure criterion of evaluation is given, and which the Skeptics, because they despise analogy, always deny” (Humboldt, 1793, p. 153).

The slow processes of plants prompted Humboldt to turn to animal physiology to advance his research on the chemical basis of living functions, or “vital chemistry”, in the *Experiments on Stimulated Muscle and Nerve Fibers* (*Versuche über die gereizte Muskel- und Nervenfaser*, Humboldt, 1797, pp. 38–40). This vital chemistry posited that phenomena of “organic substances” – being material – are rooted in the same forces and chemical processes observed in inorganic matter (ibid., pp. 40–41). In fact, during the mid-1790s, Humboldt’s views on “vital force” underwent a crucial shift. In *Florae fribergensis*, he had defined vital force as a common principle of organic beings (both plants and animals). The distinction of inanimate and animate matter depended on the differing statuses of the respective chemical processes: inanimate matter was subject to standard chemical laws,^4^ whereas animate matter was subject to vital force, defined as “the internal force that loosens the bonds of chemical affinity as well as prevents the elements of the particles from freely compounding” (Humboldt, 1793, p. 135). In the second volume of the *Experiments*, Humboldt no longer associated vital chemistry with the notion of vital force anymore.^5^ As he declared in the 1849 third edition of the popular book *Views of Nature* (*Ansichten der Natur*), “further studies in the area of physiology and chemistry” had “profoundly shaken” his views thus leading him to revise his stance (Humboldt, 1849*,* p. 310). In the *Experiments* (Humboldt, 1797, p. 433), Humboldt claimed that vital force is an “unknown force” that is “by no means proved”.

Even so, Humboldt continued to use the notion of irritability for both animal and plants: “Animal and plant fiber must, as I believe, be regarded not merely as susceptible to irritation but as continuously irritated”. In his later works, he supported an empirical account of living beings and criticized the excessive metaphysical speculations of natural philosophers about the non-chemical properties of life (see Werner, 2000).

A significant example of the alternative view can be found in the works of the psychologist and natural scientist Gustav Fechner. Fechner – inspired by Schelling’s metaphysical identity theory – defended a kind of panpsychist view of nature, and in his book *Nanna, or the spiritual life of plants* (*Nanna oder über das Seelenleben der Pflanzen*) he focused on plants and formulated several bold claims. First, he argued that plants have sensations, although these are realized in a different physiological support from that of animals**:**

“What is so peculiar in the nature of nerves that makes them alone capable of channeling psychic activity? I believe that matter in plant fibers is equally fit to this task” (Fechner, 1848, p. 40).

On Fechner’s view, plants also have instincts and drives:

“If caterpillars and spiders can feel within themselves the impulse to emit strands by means of which they attain the ends nature has given them, how could one not also accord to the toothwort an equal sentiment that presided over its upward growth?” (Ibid, p. 110).

Finally, plants also communicate by means of chemicals: “What words are to us, are to plants smells” (Ibid, p. 70). Fechner acknowledged that these claims were not grounded on sufficient evidence. Rather, they presented a general alternative between two views: the “Day view”, on which all living beings are endowed with a spiritual life, and the “Night view”, on which humans are surrounded by a mindless mechanistic world.^6^ Fechner overtly sided with the Day view: “It is difficult to think of this gushing living sphere, so full of exchanges with the inside and the outside, as devoid of sensation” (Ibid, p. 61).

Fechner’s affirmative stance concerning the Day View tipped the epistemological balance:

“Of course, we can only imagine this; we cannot directly see these soul flames of nature; but since we can imagine it, why should we not? No one forces us to open our eyes to external lights or to warm ourselves by external flames. Why do we do it? Because we like it so much better than sitting in the dark and cold. Well, we also sit in a dark and cold nature if we do not want to open the inner eye of the spirit to the inner flames of nature” (Ibid, pp. 59–60).

Fechner’s ideas were not isolated. As I will show, a comparable epistemological alternative recurs in contemporary philosophy and plant science. Given the revival of Darwinian ideas in this context, it is instructive to consider how Darwin himself situated his investigations of plants within broader theories of mind and matter.

## Darwin on evolution, materialism and panpsychism

Darwin’s intense research on plants after the publication of *The Origin of the Species* has to be contextualized in the epistemological framework of the theory of evolution and its defense. One of the main points of *The Power of Movement in Plants* was the study of plants’ daily movement, “circumnutation”. The Darwins underscored its physiological origin, based on chemical processes that direct phototropism, pointing out that this movement has no “special purpose” (Darwin & Darwin, 1880, p. 263). They granted that plants display a form of memory by association and spoke of plant “behavior” by analogy with animals: a “radicle may be compared with a burrowing animal such as a mole, which wishes to penetrate perpendicularly down into the ground” (Ibid, p. 199).^7^ Behind this analogy was the attempt to vindicate the validity of the theory of evolution in the field of botany (Whippo & Hangarter, 2009). The struggle for nourishment was not connected to purposefulness of nature, nor were plants literally endowed with either intentionality or consciousness.

Nevertheless, one may wonder whether the genealogical connection of living forms suggested to Darwin that plants might share some cognitive capacities after all. On the one hand, Darwin maintained that there is a continuous transition from instinct to intelligence in organisms. In the *Origin of Species*, he argues that “a little dose, as Pierre Huber expresses it, of judgment or reason, often comes into play, even in animals very low in the scale of nature” (Darwin, 1859, p. 208). In his notebooks, he had inclined to broaden the picture, noting:

“Instinct is a modification of bodily structure (connected with locomotion.) no, for *plants have instincts either to obtain a certain ends, & intellect is a modification of instinct* – and unfolding & generalizing of the means by which an instinct is transmitted” (Darwin 1838–39, n. 48, my italics).

This may suggest that Darwin was just ready to attribute some “low level” cognitive powers to plants. However, regarding sensitiveness, he very clearly saw the major disanalogy with animals in the absence of nerves. As the Darwins put it in *The Power of Movement in Plants*:

The most striking resemblance is the localisation of their sensitiveness, and the transmission of an influence from the excited part to another which consequently moves. Yet plants do not of course possess nerves or a central nervous system (Darwin & Darwin, 1880, p. 572).

Given this difference, contemporary physiologists conceived an alternative way of addressing consciousness by looking for its molecular basis. In this regard, Darwin’s peers were sharply divided. Thomas Huxley (1869, p. 18) argued that “thoughts are the *expression* of molecular changes in the matter of life”. His view was that conscious processes supervene on physical processes at the molecular level, although this does not entail any *explanation* of consciousness. This agnosticism was close to Darwin’s own view. In the notebooks, he had written that “the mind is function of body” (Darwin 1838–39, n. 5), but in *The Descent of Man* (1871) he pointed out that this relation does not allow us to glimpse how mind evolved in organisms:

“In what manner the mental powers were first developed in the lowest organisms is as hopeless an inquiry as how life itself first originated. These are problems for the distant future, if they are ever to be solved by man” (Darwin, 1889, p. 66).

This agnostic view was strongly different from the view of other prominent naturalists. Alfred Wallace, for example, maintained that “either all matter is conscious or consciousness is, or pertains to, something distinct from matter and in the latter case its presence in material forms is a proof of conscious beings, outside and independent of, what we would term matter” (Wallace, 1870, p. 209). Ernst Haeckel, a prominent defender of Darwinism in Germany, straightforwardly sided with Fechner’s monism. Darwin had read the *General Morphology of Organisms* (*Generelle Morphologie der Organismen*), where Haeckel presents monism, the view that matter and spirit are inseparable in whole universe – which we call panpsychism –, as the “indispensable foundation of natural science” (Haeckel, 1866, II, pp. 441–442). In numerous articles and in the widely read book *The Riddle of the Universe* (*Die Welträthsel*, 1899), Haeckel spoke of “atom-souls” and “cell-souls”, thereby granting the psychic activity of plants.

“The *plant-soul* (*phytopsyche*) is, in our view, the summary of the entire psychic activity of the tissueforming, multicellular plant […]; it is, however, the subject of the most diverse opinions even at the present day. It was once customary to draw an essential distinction between the plant and the animal, on the ground that the latter had a “soul “ and the plant had none. However, an unprejudiced comparison of the irritability and movements of various higher plants and lower animals convinced many observers, even at the beginning of the century, that there must be a “soul” on both sides. Fechner, Leitgeb, and others strongly contended for the plant-soul. But a profounder knowledge of the subject was obtained when the similarity of the elementary structure of the plant and of the animal was proved by the cellular theory, and especially when the similarity of conduct of the active, living protoplasm in both was shown in the plasma theory of Max Schultze (1859)” (Haeckel, 1923, pp. 128–129).

Against this background, Darwin sided with a kind of agnosticism (see Smith, 1978). His view can be compared to the famous claim (“*Ignorabimus*”) formulated by the physiologist Emil du Bois-Reymond in the 1872 lecture “On the limits of natural science”. According to du-Bois Reymond, consciousness in its most basic form, i.e. “sensation” (which can be identified with today’s “phenomenal consciousness”) would remain forever unexplainable. To be sure, Du Bois-Reymond was also a staunch supporter of Darwin’s theory of evolution and, in the same speech, he praised *The Descent of Man* as a work which “forces upon him [the natural scientist] the theory that the soul came into being as the result, gradually attained, of certain material combinations, and that probably, like other heritable endowments that are of use to the individual in the struggle for life, it has risen and perfected itself up to its present state through a countless series of generations” (du Bois-Reymond, 1886, p. 127). In contemporary terms, du Bois-Reymond admitted the supervenience of mental processes on neurophysiological processes and he also granted that consciousness was a natural “fact”. However, he claimed that “not only is consciousness [i.e. sensation] inexplicable by its material conditions in the present status of science, which every one will readily admit, but […] even according to the nature of things, it never can be explained by these conditions” (Du Bois-Reymond Ibid., p. 117). From this perspective, Fechner’s “Plant-” and “World-souls” were not scientific, and Haeckel’s ideas on animated atoms followed the “the spirit of a false philosophy of nature” (1886, p. 123; p. 388, 412n8).^8^

Darwin’s view was less categorical: he admitted that the origin of consciousness in non-human organisms was outside the scope of *present* science. Thus, analogy – supported by the shared genealogy of organisms in general – was the only ground for extending consciousness beyond humans. Given the absence of a nervous system, the analogy with plants was merely grounded in a kind of “behavior”. This is precisely the point from where contemporary botanists have begun to reconsider the question.

## Experiments and interpretations: a revival

The controversial status of plant cognition in contemporary botany is best understood starting from the publication of the popular book *The Secret Life of Plants* (Tompkins & Bird, 1973). In this bestseller, the authors made bold and scientifically unsupported claims about the feelings and intelligence of plants, including a musical preference for Beethoven and the capacity for mindreading. In response to early dismissive criticism of the latter claim, former CIA agent Cleve Baxter and IBM researcher Marcel Vogel replied that one had to develop empathy with plants to understand these facts. Following this episode, the scientific community developed a negative bias toward investigations into plant cognition.^9^ Nevertheless, the topic resurfaced with new research and promising results concerning plant sensation, communication, kin recognition, memory and learning.

The thesis of plant *sensation* has been recently explored by Baluska and Yokama (2021), who showed that plants are sensitive to the administration of anesthetics. This result is analogous to those that have been done with fish to prove the latter’s sentience, and similar investigations on pain markers have been conducted with insects.^10^ Overall, one implication of these studies is to bridge the gap between plants and animals.

Thus the transition from animal behavior and sentience to plants faces two problems: the first is justifying sentience beyond mammals, a topic that is itself debated (although arguments based on biological continuity and new experiments are leading to a widespread attribution of sentience beyond mammals: see Godfrey-Smith, 2021; Birch, 2024, Cf. below Sect. 6); the second is extending the analogy beyond animals. Regarding this second step, the question appears uncertain. On the one hand, it has been established that non-neural cells in both animals and plants can store information and influence biochemical responses. But these biochemical processes cannot be easily equated with animal’s sensation, since animal perception and response are adapted to their mobility and search for food (cf. Rix, 2025, pp. 33–34). Hence, some scholars talk of a peculiar “sense” in plants.

A similar point concerns plant communication. Several observations and experiments have shown that trees send chemical signals when harmed by caterpillars, fires or other physically damaging factors, thus sparking a protective reaction in other plants. Again, the controversy regards whether this can be considered as evidence of *intentional* communication (Schlänger, 2024, pp. 52–74). A connected, striking hypothesis concerns kin recognition. It has been argued that Douglas firs send nutrients to their seedlings and are capable of recognizing kin from non-kin, which could imply a kind a self-representation (Asay, Simard, Dudley 2020). Susanne Simard, among those who pioneered this line of inquiry, investigated trees to understand the way they “perceive and communicate with other plants, insects and mushrooms”, concluding that trees “cooperate” and particularly help their offspring (Simard, 2022). These views – popularized under the phrase “Wood Wide Web” – openly challenged the biological conception of life as governed by competition for survival. Simard’s insights stemmed from her discovery of the importance of biodiversity in the forestry practices. Once again, the evidence that trees connect with other tress through mycorrhizal networks is disputed in the scientific literature (Karst et al., 2023), and the anthropomorphisation of trees as communicating through the mycelium and caring for their offspring has been deemed inadequate (Jones et al*.,* 2023. Cf.; Rix, 2025, pp. 128–130).

In a striking paper, Monica Gagliano and her co-authors reported an experiment with garden peas (*Pisum sativum*), aimed at testing whether plants could undergo a kind of Pavlovian conditioning. Pea shoots were set in a Y-maze structure made of pipes, and the experiment showed that two-thirds of the shoots grew in the direction of signals associated with light, indicated by a neutral cue (fans). According to the authors, this proved a “conditioned behavior”: the plants could learn and predict the direction of a light source. “This learned behaviour prevailed over innate phototropism” (Gagliano et al., 2016).

In a critical reply, Markel (2020) reported that she had replicated the experiment but found that only 50% of the plants grew in the direction of the neutral cue. The original authors replied that the experimental setup had been different (Gagliano et al., 2020). In a recent paper, Ponkshe et al. (2024) describe their attempts at replicating Gagliano’s experiment and address the methodological issues raised by Markel, suggesting ways to facilitate further investigations. (Meanwhile, evidence of plants keeping track of visits by pollinators has added more elements to the debate on plant learning. See Mittelbach et al., 2019).

Ponkshe and her co-authors conclude that “ultimately, we need a comprehensive, comparative psychology of learning across the tree of life, respecting phenotypic comparisons to truly understand behavior’s diverse forms”. As this conclusion suggests, the epistemological issue – and controversy – at stake is broader. Gagliano’s experiment might merely prove mechanisms of associative memory in plants, without entailing any claim about sensitiveness and consciousness, but its ultimate scope is to foster investigations into further plant cognitive powers. In her 2018 book *Thus Spoke the Plant*, Gagliano maintains that plants think, feel and communicate with other plants – and even with humans – grounding this view in a mix of experimental and subjective experiential evidence.

Overall, these findings depend on a combination of new evidence and new interpretations. Their use of notions of intelligence, memory, behavior, and self, drawn from the study of animals and extended to plants, aims to question the traditional distinction among living beings. This raises two distinct issues, which are clearly illustrated by the debate over Gagliano’s results. First, there is the standard scientific need for experimental replication; second, the justification of conceptual innovation. In this latter case, the opportunity for analogy with animals has led to major controversy among scientists. A key moment has been the proposal, put forward by a group of researchers, of a new field of “plant neurobiology”. This provocative label was intended to highlight the possibility of a different realizations of cognitive functions in organisms lacking nervous systems, and to counter what they saw as a kind of “self-censorship” in contemporary science (Brenner et al*.,* 2006). Following this trend, the discipline of cognitive ecology focuses on the functional role of learning in nature, with the working hypothesis that “brains and neurons […] may not be a necessary requirement for learning” (Gagliano et al., 2014).

In a critical article, Lincoln Taiz and his co-authors noted that “regulatory interactions are occurring throughout the plant”, rather than being concentrated in the root tip. Therefore, “we could regard the entire plant as a brain-like command center, but then the brain metaphor would lose whatever heuristic value it was originally supposed to have”. The authors concluded that it is “extremely unlikely that plants, lacking any anatomical structures remotely comparable to the complexity of the threshold brain, possess consciousness” (Taiz et al., 2019).^11^

In light of this survey, the legacy of Darwin’s root-brain hypothesis – evoked in the paper on “plant neurobiology” cited above – remains controversial. The question is whether Darwin’s analogy, dismissed by 19th-century botanists, appears more plausible in a contemporary context. Certainly, the different theoretical framework may play a decisive role. As we have seen, revolutionary views on plant cognition have been inspired by theories of cognitive science such as the multiple realizability of cognitive states. Moreover, recent investigations on animal minds may have further encouraged the idea that traditional ontological boundaries can be broken. The significance of this new context will be explored in the final section.

## Concluding remarks on analogies and biases

The conceptual dimension of the controversy over plant cognition has been finely characterized by Daniel Chamowitz (2018). Given the evidence that “plants integrate many signals to adapt to their environment and increase their fitness”, Chamowitz asks: “Is this a proof of intelligence? It depends on the meaning of the word”. Those who attribute intelligence to plants define this capacity as a form of problem-solving displayed over an individual organism’s lifetime. Thus, Anthony Trewavas proposed defining plant intelligence as “adaptively variable growth and development during the lifetime of the individual” (Trewavas, 2003, p. 1). Advocates of plant cognition connect this kind of definition to the thesis that neural networks are not a necessary condition for cognitive states. After the controversial term of “neurobiology” was replaced with “signaling and behavior”, Paco Calvo restated the general claim of this approach, which.

“aims to unearth that plants *perceive and act in an integrated and purposeful manner*, and how they do it. The rationale that underlies this effort is the idea that *intelligent, flexible behavior requires coordination* among diverse plant structures. […] the field [thus] proposes an interdisciplinary and integrated view of plant signaling and adaptive behavior in order to study plant intelligence” (Calvo, 2016, pp. 1323, 1326).

In the critical paper cited above, Taiz and his co-authors (2019) protested against the resurgent “anthropomorphism” of those who talk of *learning* and *feeling* of plants. Calvo and Trewavas (2020) replied: “This is surely circular reasoning”. This exchange clearly shows the epistemological crux of the debate on plant cognition. Talk of plant “intelligence”, “learning”, “feeling” and “self-recognition” is based on an analogy with humans. The question is: How far can we meaningfully extend the analogy? A possible perspective is to reduce the issue to a matter of terminology. For example, Calvo attempted to settle the issue as follows:

“It goes without saying that plants do not share “neurons” with animals, or exhibit animal “intelligence”. If the reader wishes to keep those terms for animals exclusively, so be it. Plant neurobiology interprets plants as information-processing networks with individual cells as computational building blocks” (Calvo Garzón, 2007).

Yet this emphasis on function and computation does not entirely solve the problem. Because we use psychological terms to define human cognitive functions, the question is whether it is adequate to attribute a different kind or degree of the same functions to plants.

A similar problem has arisen in cognitive ethology. Given that definitions must start from human experience, the issue is whether it makes sense to extend the analogy to other species. In his investigation of the roots of moral capacities in primates, Frans De Waal (2016) pointed out that, in the light of evolutionary biology, “anthropomorphism” must be tempered without lapsing into the opposite mistake of “anthropodenial”, the claim of absolute human exceptionalism. The search for precedents of human morality in primates was motivated by shared biology and was, in fact, a development of Darwin’s ideas.^12^

Peter Godfrey-Smith has made a similar reasoning with respect to consciousness, starting from a critique of behaviorism:

This animal is only doing things that we know can be done unconsciously. It would then feel like nothing to be that animal. Next, this conclusion might be applied to various actual animals, whose abilities are similar to the hypothetical one. This would not be a good argument, however. The experiments the argument is based on were all done with conscious human beings, even though the people were not conscious of everything going on inside them. From these experiments we are learning that, when you are a conscious human being, a surprising amount can be done deep in the background. That does not show that everything could be in the background at once. For any normal and wakeful human being, there is something it’s like to be that person, even if much is being done behind the scenes. The same may apply to a great many animals as they make their way through the world […] The mistake that can then be made is to say: this (as studied in humans) is what experience is, and if you don’t have this going on, you can’t have experience. The alternative is that if your brain is different from ours, your experience is different, but not absent (Godfrey-Smith, 2021, ch 10).

Godfrey-Smith’s search for different kinds of minds was rooted in Darwinian ideas, particularly the view of genealogical continuity among living beings. The thesis of multiple realizability of mental states is a significant post-Darwinian element here, with the octopus serving as a model of an animal which is both very intelligent and very different from humans. This showing how the *meaning* of cognitive functions changes with the extension of the analogy beyond species that are closely related to us on the evolutionary tree. In his materialist perspective, Godfrey-Smith adds an important dimension to the logical problem of analogy: different bodily structures determine, to varying degrees, differences in mental capacities.

Given the difference between animals and plants – from the cellular level to the architecture of the body – Godfrey-Smith treats plant cognition as peculiarly distinct from all animal cases. First, he argues that nervous systems operate differently from plant biochemical signaling systems: “what nervous systems do is achieve specific kinds of cell–cell interaction—interactions that are fast, targeted, and directional” (Godfrey-Smith, 2016), while signaling in plants is massive and does not happen with fast cell–cell interactions. Second, it is difficult to define plant individuals and, hence, plant subjects:

“A plant is in some ways like a community rather than a single individual. Within a community there can be signaling and coordination; you and I might communicate closely, and still be two. Social interaction does not merge our subjectivities […].

Subjectivity is about engaging with the world as a self, and having things seem a certain way to you. Plants have less of a you. In some ways, a plant is a they, a they of stems and shoots, with signaling running between them. But that is a simplification, too; a plant is partly a community and partly an individual” (Godfrey-Smith, 2021, ch. 8).

Given these deep disanalogies, one might think the issue is merely terminological. A nominalist solution could be to define different kinds of intelligence and other cognitive powers, perhaps indicating labeled with indices (such as ‘P-intelligence’). However, this merely reformulates the problem and does not modify the epistemological balance between the attribution of non-attribution of cognitive powers to plants.

However, at least two non-scientific dimensions play a role in today’s debates.

A first point concerns ethics and law. In the case of animal minds, uncertainty about whether certain animals possess sentience and intentionality is recognized as possibly insurmountable; nevertheless, the possibility that they feel pain and pursue goals has led to proposals to attribute those capacities to them as a matter of ethical caution (Birch, 2024; Sebo, 2025).

A second point concerns different cultural views, such as those of “animist” people, who ascribe spirit or personhood to other animals and sometimes to plants and inorganic beings. These views are gaining increasing attention as sources of ecological thought and criticism of the Western ideas about society, economy and the exploitation of nature (e.g., Albert & Kopenawa, 2020; Harvey, 2006; Krenak, 2022). These perspectives can be connected to Western ecology, as well as to metaphysical views of philosophical traditions, such as Platonic animism or Fechner’s monism (Pecere, 2024a, 2024b, ch. 2; Pecere, 2024b).

These two sources of positive bias carry different weights in animal and plant science. The prospect of animal sentience and rights is strongly motivated by the intensive animal farming and medical research, whereas the idea of plant sentience and rights is usually not tied to regulating human industry. On the other hand, the idea of plant as subjects and sources of spiritual insight – drawn from Indigenous lore and experiences with psychoactive plants – is a lively factor even in science. Gagliano (2018) cites Aboriginal traditions as a source for insights into plant intelligence that predate Darwin. She argues that an “unexpected encounter” between “scientific experience and wisdom of the plants” can be crucial, recounting significant personal experiences. Similarly, Simard (Foreword to Gagliano, 2018) values the wisdom of “indigenous seers” and encounters with “plant-persons” as opportunities for learning.

Returning to Darwin, we can conclude that the epistemological alternative between a reductive view and a speculative view of plant cognition was already outlined in his time. Darwin himself occupied an agnostic position while employing a broad analogical view of behavior and cognitive powers in organisms. A significant difference in contemporary debates – besides the greater volume of empirical data – is the tendency to challenge the standard anthropocentrism of scientific notions, within a broader critique Western anthropocentrism and its ecologically destructive consequences. The epistemic scale may still be balanced, but ethical arguments encourage scientists to take analogies more seriously and even question the validity of the human standard.

## Data Availability

Not applicable.

## Footnotes

1 See the contributions of Fabrizio Baldassarri and Quentin Hiernaux in this special issue. A critical overview of pre-modern conceptions of vegetative powers can be found in Baldassarri, Blank (2021). See also Baldassarri (forthcoming).

2 For a brief outline see Pecere 2020a, 2020b, pp. 59–60. See the articles by Baldassarri and Hiernaux in this issue.

3 On Glisson and plant physiology, see Baldassarri (2024), pp. 101–105.

4 “We call that matter inactive, brute, or inanimate if its elements are combined according to the laws of chemical affinity” (Humboldt 1793, p. 133).

5 The frontispiece of this volume bears the date 1797, but it was published in 1799: see Fiedler and Leitner 2000, p. 16. Nevertheless, Humboldt writes that he found his new theory “in the year 1797” (Humboldt 1849, p. 310).

6 On Fechner’s natural philosophy see Heidelberger 2004.

7 On other analogies between animal and plant life, see Marilena Panarelli and Stella Sandford in this special issue.

8 On Du Bois-Reymond’s *Ignorabimus* see Pecere 2020b.

9 See the brief account of this precedent and its impact in Schlänger 2024, pp. 13–16.

10 On fishes see, e.g., Sneddon et al., 2014. For an outline of these investigations see Dill and Sebo 2025.

11 It must be pointed out that this conclusion rules out that the multiple realization of mental states could allow identifying interactions “occurring throughout the plant” to a physical substrate of consciousness, while it does not entirely rule out the validity of the brain analogy.

12 “Any animal whatever, endowed with well-marked social instincts, the parental and filial affections being here included, would inevitably acquire a moral sense or conscience, as soon as its intellectual powers had become as well developed, or nearly as well developed, as in man» (Darwin 1889, p. 98). Darwin, in turn, was inspired by Hume’s application of Newtonian analogy to the study of animals: “Tis from the resemblance of the external actions of animals to those we ourselves perform, that we judge their internal likewise to resemble ours; and the same principle of reasoning, carry’d one step farther, will make us conclude that since our internal actions resemble each other, the causes, from which they are deriv’d, must also be resembling. When any hypothesis, therefore, is advanc’d to explain a mental operation, which is common to men and beasts, we must apply the same hypothesis to both” (Hume 2007).
